# SNG and DNG meta-absorber with fractional absorption band for sensing application

**DOI:** 10.1038/s41598-020-69792-4

**Published:** 2020-08-04

**Authors:** Ahasanul Hoque, Mohammad Tariqul Islam, Ali F. Almutairi, Muhammad E. H. Chowdhury, Md. Samsuzzaman

**Affiliations:** 1 Department of Electrical, Electronic and Systems Engineering, Faculty of Engineering and Built Environment, Universiti Kebangsaan Malaysia, 43600 Bangi, Selangor Malaysia; 2 Electrical Engineering Department, Kuwait University, 13060 Kuwait City, Kuwait; 3 Department of Electrical Engineering, Qatar University, 2713 Doha, Qatar; 4 Faculty of Computer Science and Engineering, Patuakhali Science and Technology University, Patuakhali, Bangladesh

**Keywords:** Electronic structure, Electrical and electronic engineering

## Abstract

This paper reports on a tunable transmission frequency characteristics-based metamaterial absorber of an X band sensing application with a fractional bandwidth. Tunable resonator metamaterial absorbers fabricated with dielectric surface have been the subject of growing attention of late. Absorbers possess electromagnetic properties and range modification capacity, and they have yet to be studied in detail. The proposed microstructure resonator inspired absorber with triple fractional band absorption consists of two balanced symmetrical vertical patches at the outer periphery and a tiny drop hole at two edges. Experimental verification depicted two absorption bands with single negative (SNG) characteristics for two resonances, but double negative (DNG) for single resonance frequency. The mechanism of sensing and absorption was analyzed using the transmission line principle with useful parameter analysis. Cotton, a hygroscopic fiber with moisture content, was chosen to characterize the proposed absorber for the X band application. The electrical properties of the cotton changed depending on the moisture absorption level. The simulation and the measured absorption approximately justified the result; the simulated absorption was above 90% (at 10.62, 11.64, and 12.8 GHz), although the steady level was 80%. The moisture content of the cotton (at different levels from 0 to 32.13%) was simulated, and the transmission resonance frequency changed its point in two significant ranges. However, comparing the two adopted measurement method and algorithm applied to the S parameter showed a closer variation between the two resonances (11.64 and 12.8 GHz) which signified that a much more accurate measurement of the cotton dielectric constant was possible up to a moisture content of 16.1%. However, certain unwanted changes were noted at 8.4–8.9 GHz and 10.6–12.4 GHz. The proposed triple-band absorber has potential applications in the X band sensing of moisture in capsules or tablet bottles.

## Introduction

The dielectric properties of natural materials are well established, and applying engineering techniques to these materials has produced several innovative products. The artificially engineered materials, *metamaterials*, is a developing research field and has attracted significant interest among scientists and researchers. Unique and unconventional dielectric properties have been demonstrated in a naturally accessible material using an engineering tactic named *meta (beyond)-material*. Immense strategic interest has directed researchers to versatile applications, such as super-lenses, invisibility cloaking, plasmonic polarization, optical bent black holes, electromagnetic absorption, and solar energy harvesting. A two dimensional metamaterial, which is sometimes referred to as a single-layer metamaterial with a sub-wavelength scale, has the advantage of low-profile geometry and excellent impedance match capability. Microwave metasurface absorbers generally provide high impedance surface (~ 377Ω) at the substrate-air layer, and substrates are usually a lossy dielectric material, which expedites the loss during wave propagation^1–3^. Wideband microwave absorbers are typically made from materials with magnetic dipole or solid dielectric properties. The operating frequency is preserved with appropriate medium parameters (i.e., permittivity, permeability), although obtaining such properties is always challenging. Fortunately, extensive study of absorbers has revealed that an array of the absorbers could modify the effective material properties with narrow absorption bandwidths. Therefore, diverse techniques and designs have been proposed to enhance the bandwidths, such as modifying the resonator structure, lumped elements resonators, layered structure, and frequency selective surface (FSS), among others^4–7^. High absorption and narrowband responses can clarify some design variations with a closed-ring shape, octagonal shape, resistive-load imposing, and some ring variations^8,9^. Unfortunately, higher ohmic loss and bandwidth constraints persist on the suggested unit cell, rendering them unsuitable for microwave applications.

As a result of technical benefits such as their miniature size, low-cost, simple fabrication process, low weight, and most importantly, high absorption, sub-wavelength metamaterials have found wide applications in microwave regimes^10–13^. Furthermore, at a specific resonant frequency, split-ring resonators (SRRs) can provide negative permittivity (ε) and permeability (µ) and are particularly useful in dispersive sensory applications^14^. For example, SRR loaded on a silicon substrate has been suggested for high strain tolerance, while resonance frequency shifting could be useful for sensing mechanical deformation^15^. Transmission and reflection coefficient-based sensing by metamaterial sensors is entirely a change of resonance frequency/wavelength or Q-factor. From 2007 onwards, several metamaterial resonator structures have been used for different sensing applications, such as bulk dielectric material characterization for composition, water or moisture content, and food quality^16–19^. The samples considered in such research were one millimeter in size or more. Thick dielectric materials such as FR4, wood, Rogers, and Taconic have been used for structure capacitive characteristics enhancement, as they modify the permittivity and permeability of the overall unit cell. Based on the structure of the unit cell resonator and material properties showed significant resonance frequency shifting. However, this method of measuring necessitates covering up the entire structure by the sample, which is considered a substantial limitation because the sample quantity modifies the capacitance of the overall structure in the experiment.

The proposed microstructure resonator metamaterial absorber (MRMA) maintains a balance between the absorption and sensing ability. Thus, though the sample position changes during the experiment, it does not affect the capacitance of the intended resonance frequency range; a new metamaterial-based absorber is added to the resonator and the application to measure the moisture content in cotton slabs. The absorption performance varied, showing 80% (with two resonance) according to the measurement and was above 90% (three resonance) during the simulation for X band application. Furthermore, the high absorptivity was maintained by more than 90% (single resonance point), with a wide incident angle of up to 120° for both transverse electric (TE) and transverse magnetic (TM) for single resonance frequency.

## Results

### Design and methodology

**Figure 1:**
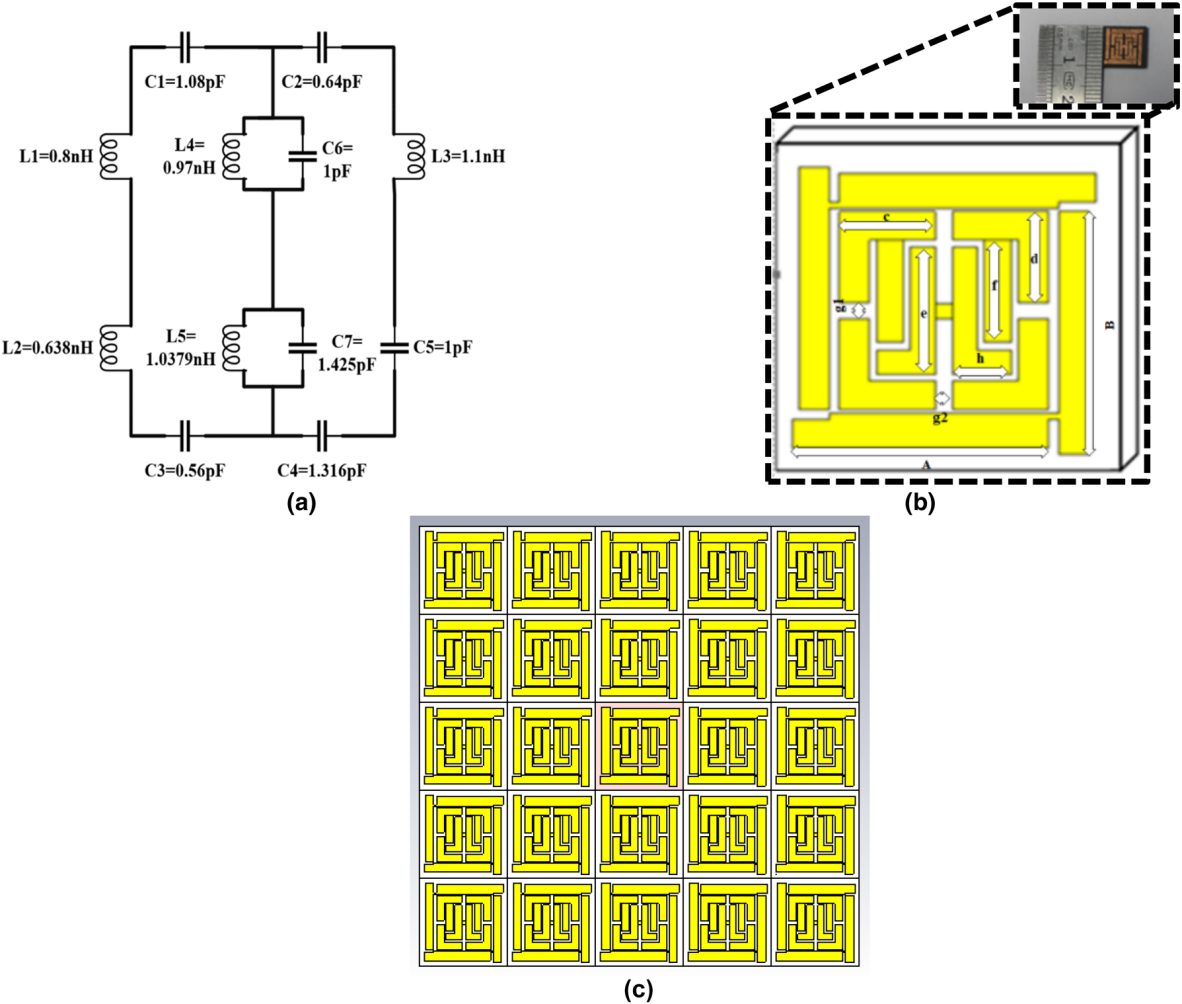
(**a**) Equivalent circuit (**b**) MRMA unit cell with major dimensions (fabricated cell inset) (**c**) 5 × 5 array design.

The electromagnetic (EM) properties of a medium or material are defined by the key parameters of permittivity (*ε*) and permeability (*μ*). During plane wave propagation, a split of two wave components, transmitted and reflected waves, occurs, depending on the incident angle and discontinuity of dielectric material results. The incident angle leads to an increase in the number of wave components. However, the metamaterial structure exclusively modifies the dielectric parameters. Therefore, focusing on these parameters one by one can mathematically extract the effective medium characteristics for the proposed structure. The magnetic and electric fields for effective medium parameters follow Eqs. (1) and (2), rather than considering the conventional field equation^18^.

1 $$ B_{ave} = \mu_{eff} \mu_{0} H_{ave} \;{\text{and}}\;D_{ave} = \varepsilon_{eff} \varepsilon_{0} E_{ave} $$

where $B_{ave}$ is the average magnetic flux density, $\mu_{eff}$ is the effective permeability of the medium, $\mu_{0}$ is the Free space permeability, $H_{ave}$ is the average magnetic field density, $D_{ave}$ is the average electric flux density, $\varepsilon_{eff}$ is the effective permittivity of the medium, $\varepsilon_{0}$ is the free space permittivity, and $E_{ave}$ is the average electric field intensity. In Maxwell’s equation (integral form) to relate flux densities

2 $$ \int\limits_{C} {H.d{\text{I}}} = 0 + \frac{\partial }{\partial t}\int {\int\limits_{S} {D.d{\text{S}}} } \;{\text{and}}\;\int\limits_{C} {E.d{\text{I}}} = 0 - \frac{\partial }{\partial t}\int {\int\limits_{S} {B.d{\text{S}}} } $$

each parameter is considered around the surface as *H* = the magnetic field density, *B* = the magnetic flux density, *E* = the electric field intensity and *D* = electric flux density. The integral function is calculated along the surface of a unit cell or structure when the magnetic field shows an inhomogeneous variation with rapid changes after an electromagnetic wave propagates through it. Generally, permittivity becomes unity in homogeneous field distribution, but in this scenario, H and B are relatively different, as both are asymmetric and inhomogeneous along the resultant EM wave propagation. Therefore, the $\mu_{eff}$ is significantly altered. The following sections illustrate the resonator magnetic field variation as a change of a valid parameter. In this paper, the dielectric properties (*ε*, μ) of the microstructure resonator are derived from the traditional procedure of obtaining material parameters from transmission/reflection (TR) data^20,21^. However, the substrate dielectric property and patch structure equivalent LC tank circuit both affect the complex permeability as well as the complex permittivity. The unit cell size in the subwavelength dimension creates an inequality in terms of recognizing the stable spatial dispersion; instead, weak spatial dispersion is considered. In relation to the composite media and polarization dependency, if we completely ignore the product of wave vector (*k*) and patch layer particle dimension (d) still for a general idea of homogenous field distribution for any extraordinary wave^22^.

3 $$ \left\{ {\frac{\partial }{{\partial x^{2} }} + \frac{\partial }{{\partial y^{2} }} + (k^{2} - \beta_{z} )} \right\}E_{z} = 0 $$

where the wave is traveling along the *z-*direction, $\beta_{z}$ is the phase constant with the boundary condition of E-field to propagation is null. Here, the wave vector must satisfy, $\sqrt {k^{2} (\beta_{x} ,\beta_{y} ,0) + \beta_{z}^{2} }$, which is incompatible with normal wave propagation conditions. However, the microstructure resonator permittivity (specifically the axial component) can be determined from $\varepsilon (k,\beta_{z} ) = \varepsilon_{h} (1 - \frac{{k_{p}^{2} }}{{k^{2} - \beta_{z}^{2} }})$. Here $\varepsilon_{h}$ is the permittivity of the substrate or host medium and $k_{p}$ is the artificial frequency formed by the patch structure and medium. The proposed metasurface absorber is designed based on the transmission line principle with a qualitative comparison of various microstrip lines, modeled by the equivalent circuit shown in Fig. 1^23^. This circuit was simulated in the Advanced Design System (ADS) software for the primary justification of the S-parameter. The L1 to L3 and C1 to C4 lumped elements correspond to the outer layer patch. The transmission line parametric values (Fig. 1a) are optimized by the software. The two back to back tank circuit was adjusted, which represents the inner microstrip patch, and the simulated transmission, and reflection parameter (real) value are plotted in Fig. 6c, along with the CST Microwave studio simulated and measured values. In addition, consider a microstrip line with an inhomogeneous transmission line since strip lines are not entirely contained in substrate and propagation, not purely TEM. The fabricated unit cell design is an extension of earlier research that combines the proposed MRMA^24^. Here, there are two additional vertical patches with a tiny patch bridge and rectangular drop hole at the edges. As stated, previously, the unit cell maximum absorption has been found to be between 8.35 and 8.64 GHz, whereas the new MRMA with sensing characteristics shows a triple absorption of above 90%^25^. Furthermore, the MRMA shows strong negative permeability, also known as mu-negative (MNG) on those bands^25^. However, in terms of the simulation and measurements, the proposed sensing unit showed differences in its absorption parameters. The subwavelength dimension of the unit cell was 0.14 × 0.14 × 0.014 λ at 10.62 GHz and was designed by the Finite Integration Technique (FIT).

The unit cell was designed on a Rogers RT5880 substrate of 1.575 mm thickness with a 0.035 mm copper patch layer. RT5880 is a glass microfiber reinforced PTFE composite aligned for exacting strip lines and microstrip high-frequency circuit applications. The proposed unit cell long microstrip lines at two edges are responsible for the resonance and are not symmetrical. Therefore, a current loop can form to show a balanced dipole moment and temporary magnetic field. Furthermore, a low dissipation factor and wide thickness availability make this substrate more suitable to the proposed microwave sensing application. The ground plane is eliminated from the unit cell and is replaced by a conductive copper plane, and the three layers are bonded by double sided tape, forming an overall sandwich structure. Table 1 depicts the physical measurement of the proposed microwave sensing unit cell.

**Table 1:** MRMA unit cell dimension.

Parameter	*A*	*B*	*c*	*d*	*e*	*f*	*h*	*g*_*1*_	*g*_*2*_
Size (mm)	7.60	7.60	2.85	2.85	4.00	3.20	1.75	0.5	0.5

As cotton is an anisotropic material, it is necessary to measure its electrical properties in free-space measurements. Anisotropic media shows *birefringence*, where a single incident wave enters from free-space to the boundary of the medium. Later an output of two refracted waves or a single incident wave from an anisotropic to a free space medium, leaves two reflected waves^10^. This results in a second-order polynomial for wave vector (*k*) in the propagation direction, which is only possible when the single ordinary wave has a positive and negative propagation value. This means that the second-order differential dispersive equation in both the E and H field yield the following solutions. This unbounded anisotropic media is restricted by three-dimensional radiation condition^26^.

5 $$ E_{z} (x,y,z) = E_{0} e^{{ - j(k_{x} x + k_{y} y + k_{z} z)}} $$

6 $$ H_{z} (x,y,z) = H_{0} e^{{ - j(k_{x} x + k_{y} y + k_{z} z)}} $$

**Figure 2:**
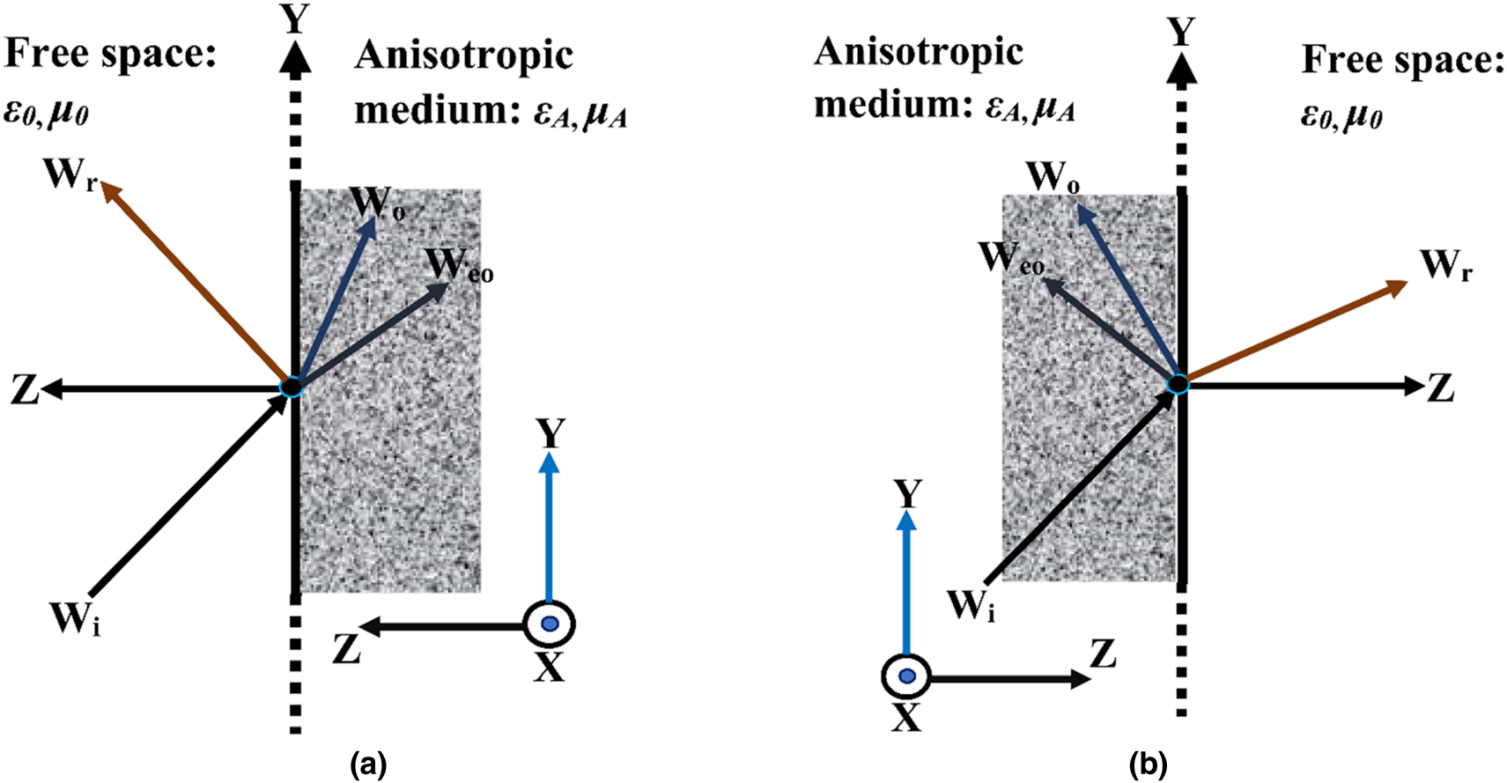
Plane-wave incident and scattering of wave propagation component (**a**) from free space to anisotropic medium (**b**) from anisotropic to free space.

Further solutions (using Eqs. (5) and (6)) give a fourth-order polynomial equation for an anisotropic medium whose roots yield the four values of *k*_*z*_ (wave vector along the z-direction shown in Fig. 2). These roots represent the ordinary (**W**_**o**_) and extraordinary (**W**_**eo**_) waves, and the terminology of the signs is the propagation direction. **W**_**i**_ and **W**_**r**_ symbolize the incident and reflected waves, respectively. The free space and anisotropic medium permittivity and permeability are denoted by *ε*_*0*_, *µ*_*0*_, *ε*_*A*_, *µ*_*A*_ respectively. The transmission and reflection from the anisotropic medium are likely to extract the electrical properties.

### Discussion

**Figure 3:**
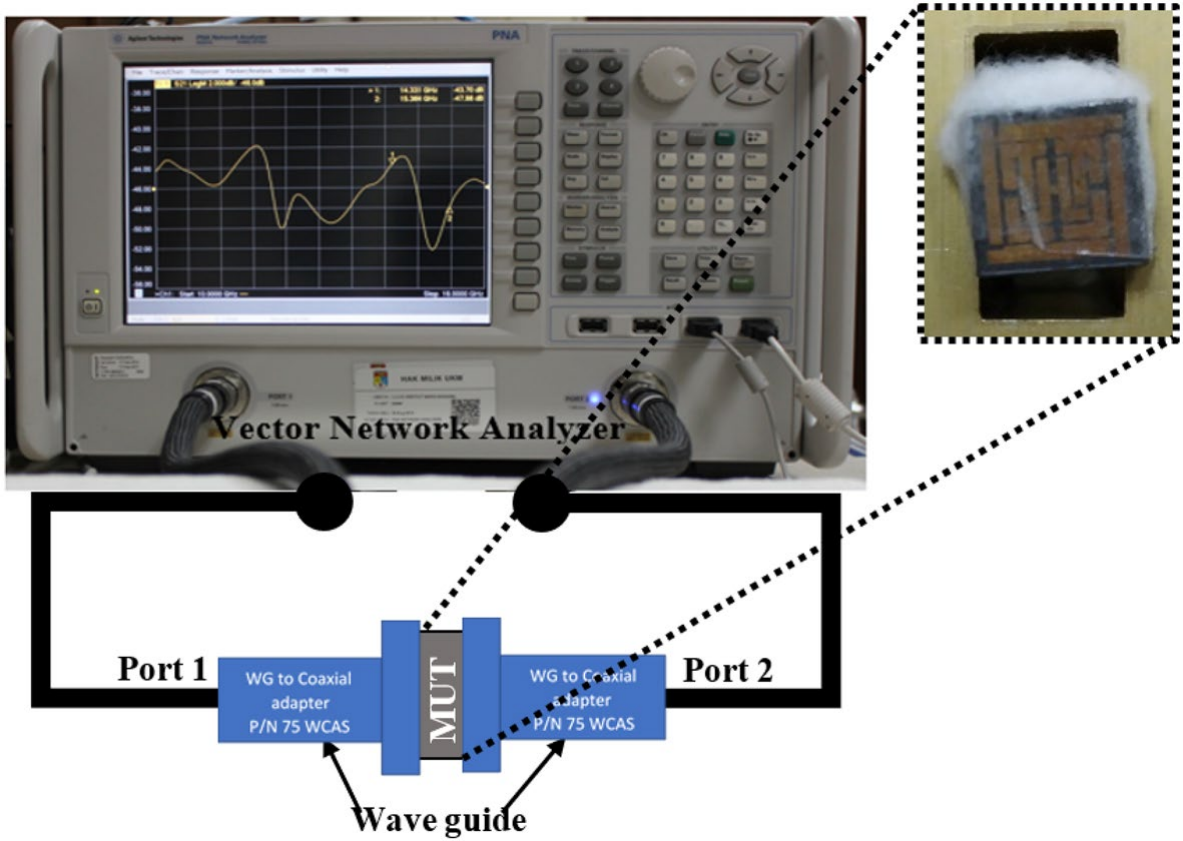
Experimental setup to measure the absorption of the proposed absorber.

The experimental setup for measuring the absorption ratio of the proposed absorber with the cotton slab between the two-unit cell is shown in Fig. 3. Waveguide ports connected to the vector network analyzer (VNA) held the unit cell so wave propagation can penetrate unit cells with cotton. The VNA Agilent N5227A was used to measure the S-parameters. The absorption ratio (A) was defined as $A(\omega ) = 1 - T(\omega ) - R(\omega )$ where T(ω) and R(ω) represented the transmission and reflection coefficients, respectively. T(ω) and R(ω) could be calculated from the insertion loss (S_21_) and return loss (S_11_), respectively. As the conductor covers the backside of the absorber entirely, we could calculate the absorption ratio by measuring only the reflection coefficient. A higher absorption efficiency of a metal-backed absorber could be achieved by minimizing the reflectance $\left| {S_{11} } \right|^{2}$ of the absorber. During the normal incidence of the EM wave, the reflection coefficient can be given by,

6 $$ \Gamma (\omega ) = \left| {S_{11} } \right|^{2} = \frac{{Z_{MRMA} (\omega ) - Z_{0} }}{{Z_{MRMA} (\omega ) + Z_{0} }} $$

The EM wave impedance in the free space was *Z*_0_ = 377Ω, and the metasurface unit cell absorber was $Z_{MRMA} (\omega ) = Z_{0} \sqrt {\mu_{MRMA} (\omega )/\varepsilon_{MRMA} (\omega )}$. At the interface of the unit cell, the expected reflection $Z_{MRMA} (\omega )$ should be unity.

To avoid interference from other undesired electromagnetic waves, the material under test (MUT) was placed close to the two waveguides, as shown in Fig. 3. A time gating function from the network analyzer was used to ensure the measured signal reflected from the proposed absorber^27^. The right-angle adapter had dimensions of 38 × 38 × 30 mm with a rated theoretical insertion loss of 0.25 and VSWR 1.25. The guide opening dimension was 19.05 × 9.52 mm for the wave propagation. The wave passed through the waveguide port and interacted with the MUT.

**Figure 4:**
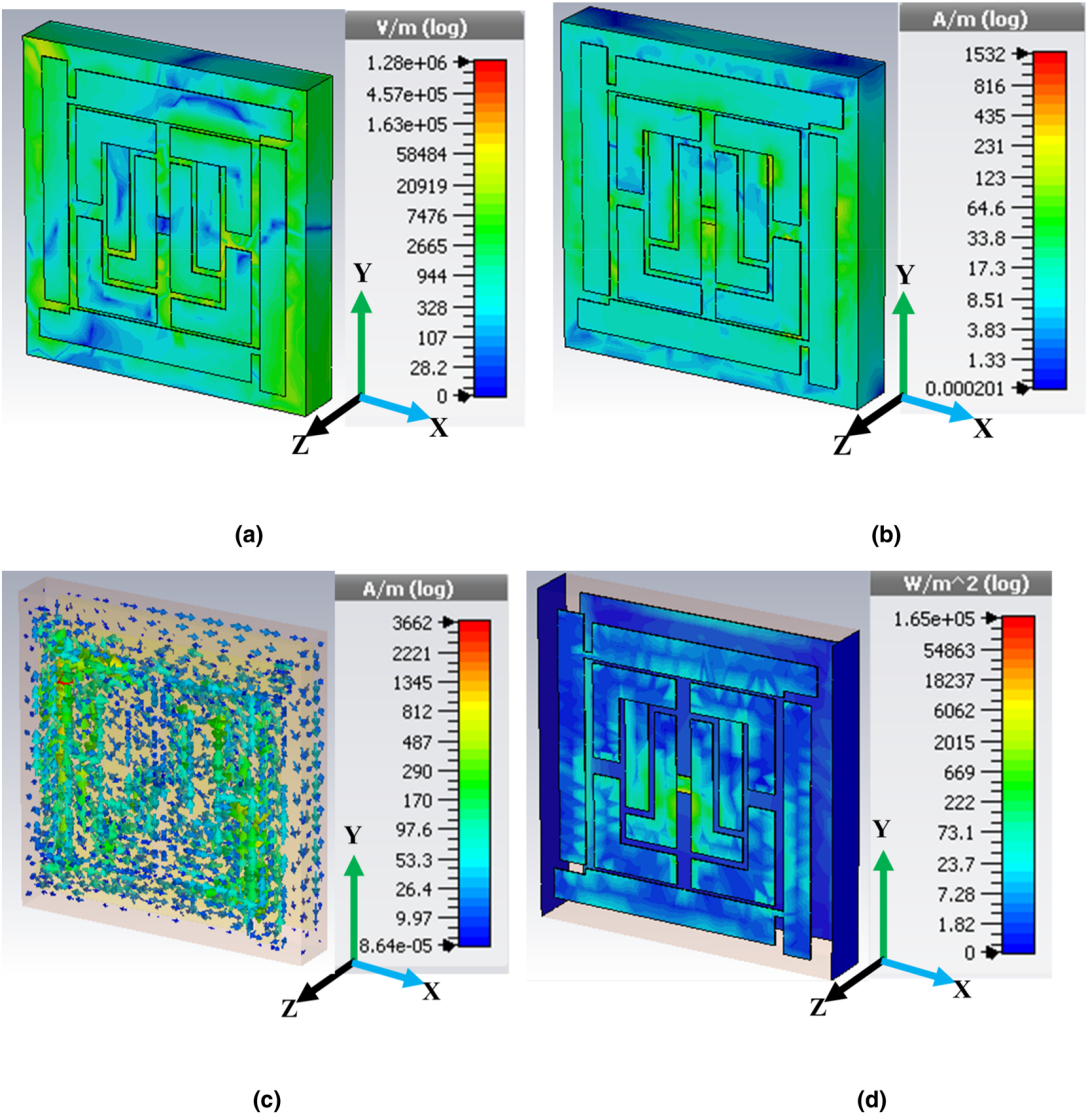
Distribution of (**a**) electric field, (**b**) magnetic field, (**c**) surface current and (**d**) power loss density at 11.94 GHz (second resonance point arbitrarily).

As the EM wave penetrated the unit cell, the parametric response showed triple resonance in simulation, which was evident in the transmission and reflection coefficient. Mathematical and physical explanations in this section using the spatial dispersion phenomenon are useful in understanding these results. The proposed MRMA substrate stood with a multiplier and eventually, on most field components (either the E-field or H-field), could not dominate the propagation. Furthermore, the equivalent circuit model (Fig. 1a) explains that dominating the inductive and capacitive component exists on the edges of the unit cell. In particular, the connected edge patch created (with a small drop hole) a steady charge carrier path to show a strong electric field (Fig. 4a). Multiple slotted patches were responsible for the other responses shown in the absorption of the unit cell. A similar approach to the H-field (Fig. 4b) at the same resonance frequency depicted an approximately invariant field distribution. The *Helmholtz* equation for the homogenous field in a bi-anisotropic resonator significantly radiated the field and, therefore, there was less consumption of the H-field component. Moreover, a mutual coupling of patches moderately accepted the individual field at the center of MRMA, which accelerated resultant H-field orientation. In Fig. 4c, the surface current distribution at the resonance frequency (11.94 GHz) clearly indicated that the transmission line component in the microstrip form had a significant effect. Vertical edge patches (with a drop hole of 0.30 mm) in both sides of the unit cell (A = B = 7.60 mm, width = 0.90 mm) was optimized for resonance. Close observation of the reflection (S_11_) coefficient demonstrated that the currents on the dominating microstrip line conductors were antisymmetric at the resonance. Hence, a current loop was created that can be described by an equivalent magnetic dipole moment. This moment is responsible for the artificial magnetism of the unit cell. Such a resonance was referred to as magnetic resonance, as shown in Fig. 4b. The power loss density of the proposed absorbing unit is shown in Fig. 4d at the same resonance frequency.

**Figure 5:**
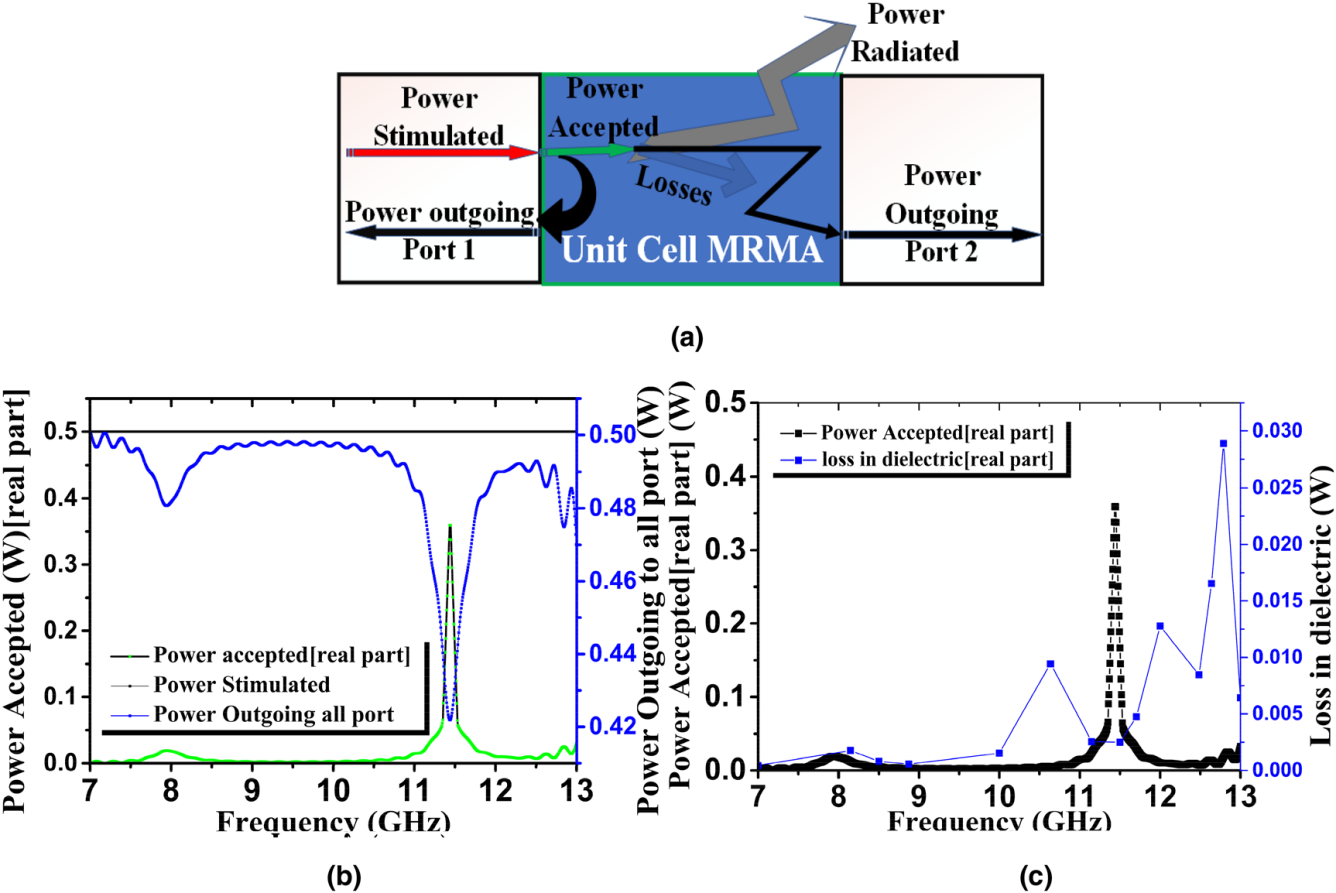
Power distribution in unit cell MRMA (**a**) schematic diagram of power balance, (**b**) frequency versus power distribution for single port excitation (**c**) loss in dielectric substrate versus power accepted.

Details of the power loss in the unit cell are discussed in Fig. 5. At 11.94 GHz, most of the cell losses amounted to 1.83–23.7 W/m^2^ (log scale), while the maximum occurred around the tiny patch bridge of the microstructure resonator. The degree of power flow was dense in the transmission line region, as the electric field was fed to the transmission line. Following this, it tended to reduce in the patch aperture area as distance increase from the feed increased. At the center of the unit cell, the feeding amount was stronger than the peripheral patch.

EM field numerical analysis was performed in the power flow through the unit cell using the following principle (Fig. 5a); power simulated = power accepted + power outgoing (all ports). It is noteworthy that the simulation and measurements were both performed on the two (2) ports method; therefore, when referring to *all ports*, we are also referring to the two (2) ports. The simulated power at any port is divided into accepted and outgoing power in the unit cell. Here, outgoing power represents all associated power that left the unit cell using those ports. Furthermore, the accepted power exchanged into losses, such as in dielectric materials, copper patches or in transmission line lumped components. For antenna applications such as transmitting and receiving, biomedical or remote sensing, this accepted power signifies the radiated power. In Fig. 5b, the proposed absorber accepts maximum power of 0.35 W at 11.43 GHz although several lossy parameters are associated with it. The dielectric loss versus the accepted power in the unit cell (Fig. 5c) was the degree of the displacement current in the patch to the substrate^28^. Generally, the plane wave propagation was dispersive. Therefore, the complex permittivity of the material at a lower frequency was marginal, though it was higher at higher frequencies. Accurate modeling of dispersive phenomena would reduce this loss. However, the dielectric loss modeled by *Debye and Lorentz*, directly states the frequency dependency of the real and imaginary part of permittivity $\varepsilon = \varepsilon^{\prime}(\omega ) - j\varepsilon^{\prime\prime}(\omega )$. Furthermore, loss may occur due to the uncertainty of MUT and other EM energy sensitive objects around the near field that are not adequately controlled. In every active excitation in a port, the stimulation of the power is measured from the signal generator to the port. The sum of all stimulated powers per port is known as *power stimulated*. In a waveguide port, the amplitude of the power depends on the mode. For *N* excitation modes, the stimulated power at the port is

7 $$ P_{s} (port) = \frac{1}{2}\sum\limits_{n = 1}^{N} {(A)_{n}^{2} } $$

where the *P*_*s*_ (*port*) is the power stimulated at the port, *A* is the amplitude of this time-harmonic excitation for the ports, and the plane waves are constant across all frequencies (typically 1). The proposed unit cell simulated power was 0.5 W, according to the simulation set-up. Figure 5b shows that the stimulated power was unchanged over the frequency spectrum. The *real part* of complex average power (*P*_*CA*_), which is applied to the unit cell in a *Z*-direction (positive), is given by

8 $$ P_{CA} = {\text{Re}} \left\{ {\frac{1}{2}\int\limits_{A} {\vec{E} \times \vec{H}} .zdz} \right\} $$

Equation (7), also valid for port 2 (negative *Z*-direction). It describes the net energy flow into the unit cell at a specific port. Using a simulated mode (TE_11_ and TM_11_) in the proposed unit cell waveguide port, and calculating each mode, hence accepted power per port is calculated by the software. Here, the power flow in the unit cell at a resonance frequency of 11.94 GHz was plotted for convenience, and a similar procedure was applied for the other two resonances (10.62 and 12.8 GHz).

**Figure 6:**
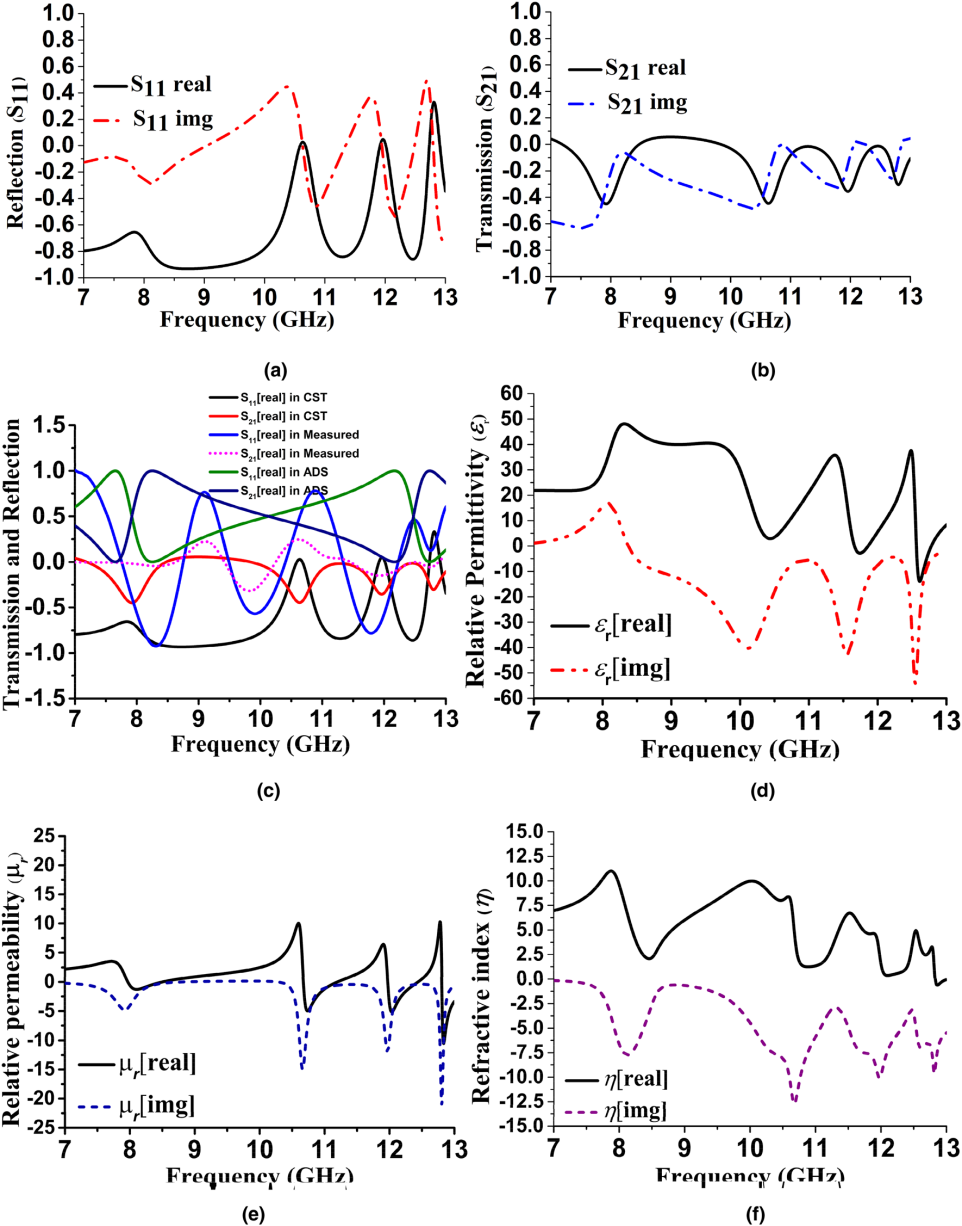
Reflection, transmission, and dielectric properties of MRMA in simulation (**a**) reflection characteristics, (**b**) transmission characteristics, (**c**) reflection versus transmission (real part) in simulation (CST, ADS software) and measured results, (**d**) relative permittivity (**e**) relative permeability and (**f**) refractive index using the Direct Refractive Index (DRI) method.

After completion of the design, the boundary condition was imposed on waveguide port 1 and 2 without enforcing any polarization angle. Standard conditions were kept for permittivity and permeability, thermal and electrical properties to optimize the result. Furthermore, the electric field applied to the X-axis, magnetic field to the Y-axis, and Z-axis kept as open space for field propagation. Scattering parameter reflection coefficient (S_11_) and transmission coefficient (S_21_) were analysed for field interactions. Figure 6a–c shows the transmission and reflection characteristics in the simulated spectrum. The real and imaginary parts of the S_11_ and S_21_ presented over the X band with a triple resonance point. The relationship from E.J. Rothwell was used in the Nicolson-Ross-Weir (NRW) method to calculate the refractive index^20^

$$ S_{21} = js_{0} S_{11} M\;{\text{where,}}\;s_{0} = \pm 1\;{\text{and}}\;M = \sqrt {\frac{{1 - \left| {S_{11} } \right|^{2} }}{{\left| {S_{11} } \right|^{2} }}} $$

The above relationship depicts an unchanged magnitude with polarity change; consequently, the traveling EM wave in a unit cell gave no identical value, despite the shift in the polarization angle from − π to + π. A metamaterial property was expected of the fluctuating points. Table 2 provides the characteristics.

**Table 2:** Metamaterial characteristics of MRMA.

Resonance frequency (GHz)	Permittivity (ε)	Permeability (µ)	Refractive Index (η)
10.62	6.82	− 0.612	4.27
11.64	− 0.883	2.12	5.87
12.8	− 1.173	− 4.80	− 0.327

It is known that the dielectric constant of a typical material is to store electrical energy, and the tangential loss is electrical energy loss due to heat. Therefore, the imaginary part of a relative permittivity (*ε*_*r*_) curve is associated with dielectric losses, and the real part represents the degree of polarization. In Fig. 6d, the higher-value polarization led to an excellent *ε*_*r*_ value. Furthermore, as a frequency-dependent parameter, the dielectric constant was inversely proportionate to polarization, but this relationship became void in a rapidly changing field. A positive imaginary part corresponds to energy absorption and is measured according to the charge of a capacitor by placing a dielectric between the conductors or electrodes. Measuring the *tan δ* is another way of calculating the imaginary part. In a general $Z$-the measured impedance of a parallel plate capacitor containing MUT.

$h$-the thickness of the MUT.

$S$-are size or dimensions of the MUT.

$\varepsilon_{0}$-permittivity of vacuum.

$$\omega$$-angular frequency.

$$j$$-imaginary unit.

$$C$$-capacitance.

$\varepsilon_{r} = \varepsilon^{\prime}_{r} - j\varepsilon^{\prime\prime}_{r}$-complex permittivity (dielectric constant)

9 $$ C = \varepsilon_{0} \varepsilon_{r} S{/}h $$

10 $$ Z = 1/j\omega C $$

**Figure 7:**
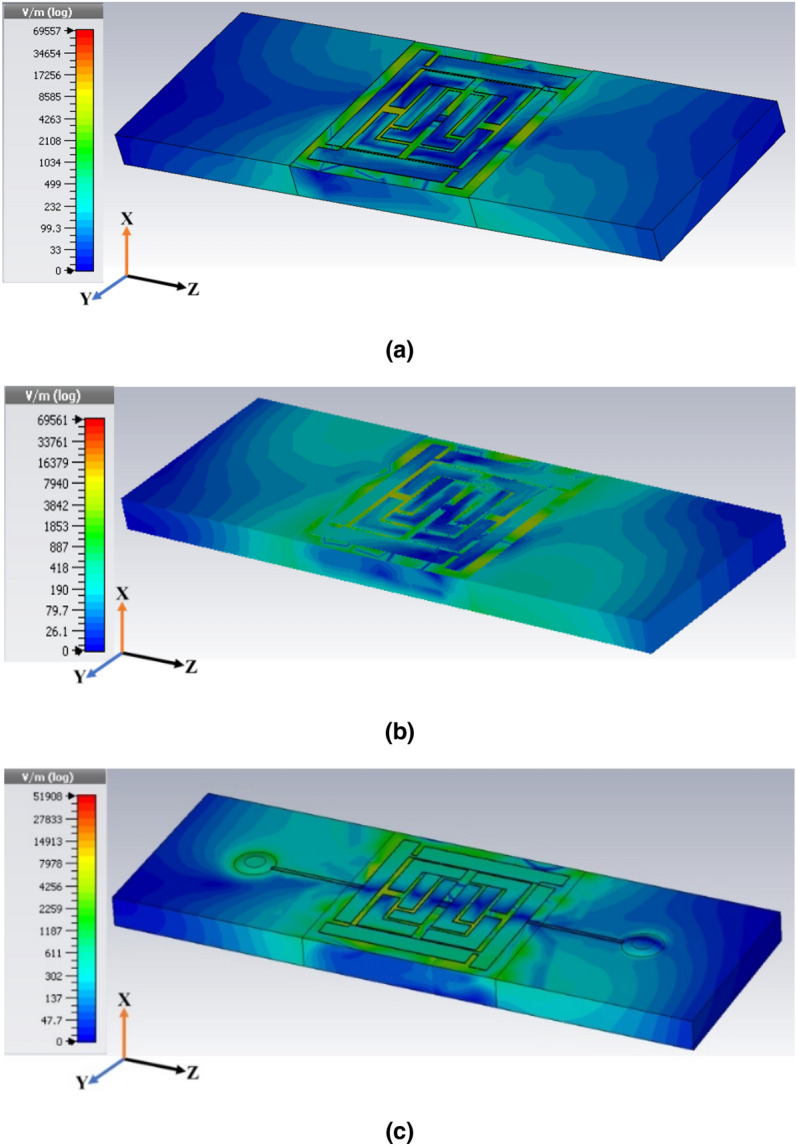
Numerical E-field intensity distribution for DNG characteristics during 12.8 GHz resonance (**a**) TE mode (**b**) TM mode and (**c**) connecting circular patch effect for DNG from free space to unit cell.

It is evident from Eqs. (9) and (10) that, C and Z will become complex since $\varepsilon_{r}$ is a complex quantity. Having known the Z and dimensions of the capacitor, the $\varepsilon_{r}$ can easily be determined. Notably, a simplified model is valid if (1) the capacitor has a negligible fringing capacitance, (2) there are no air gaps between metal the electrodes and MUT, and (3) the frequency is sufficiently low to neglect parasitic inductances. Moreover, the dielectric constant in a harmonic model states that the static dielectric constant at low frequencies is independent of the frequency^29^. However, during EM field propagation, when there are rapid changes in the field from low to high frequency, most of the materials act somewhat like plasma oscillation. In a graphical representation of the real part of the dielectric constant, such a state should smoothly approach a constant value (for low frequency), whereas the imaginary part should approach zero. High frequencies should begin as negatives and should gradually slope up to 1, while those that are imaginary should move toward zero. The dispersion of electromagnetic waves has been reported on in previous literature on dielectric performance^30,31^. However, an unavoidable limitation (Fig. 6e) is the non-zero relative permeability (*µ*_*r*_) of the real part. This means that an inherent spatial dispersion exists in all three resonance frequencies, and the deviation between equivalent and effective permeability ($\mu_{eff}$) is too large. Therefore, non-physical negative dispersion has a finite value, which ends up close to 1 at resonance. Another metamaterial characteristic is the refractive index (*η*), which is expected to be negative as per the MRMA design structure, as well as the impact of Eqs. (1) and (2). Combining the negative relative permittivity with the negative permeability (Fig. 6d, e) of the unit cell results in a metamaterial with a negative refractive index (Fig. 6f), provided that the negative values of the permittivity and permeability coincide at a specific range of frequencies. The direct Refractive Index (DRI) is a method for extracting this property from the S-parameter^32^. Table 2 shows *η* =  − 0.327 in the subwavelength dimension of the unit cell is the potential for the proposed application. The proposed unit cell simulated in the FIT method used the boundary condition (electric field in X-axis and magnetic field in Y-axis) through the waveguide port periodically for numerical analysis. Though the proposed MRMA unit cell shows strong DNG at 12.8 GHz, the two resonance frequencies are not significant or shows SNG property. To understand this better, we observed the electric field (absolute value) distribution and wave propagation characteristics by laying two blocks of air (Fig. 7) as a free space medium. The dimensions of the slab were similar to the unit cell, and the single block to the multi-layer field was numerically studied in TE and TM mode. Furthermore, propagation characteristics were considered to identify the behavior with finite medium conditions^33^. It can be assumed from Fig. 7a, b, that 12.8 GHz EM propagation was permissible, since *ε* and *μ* were both negative, though they were not strong or highly negative. As a result, the evanescent field still remained in that region. Despite using a circular connected patch in Fig. 7c, a field propagation near the patch proved that the refractive index was slightly negative on that point.

**Figure 8:**
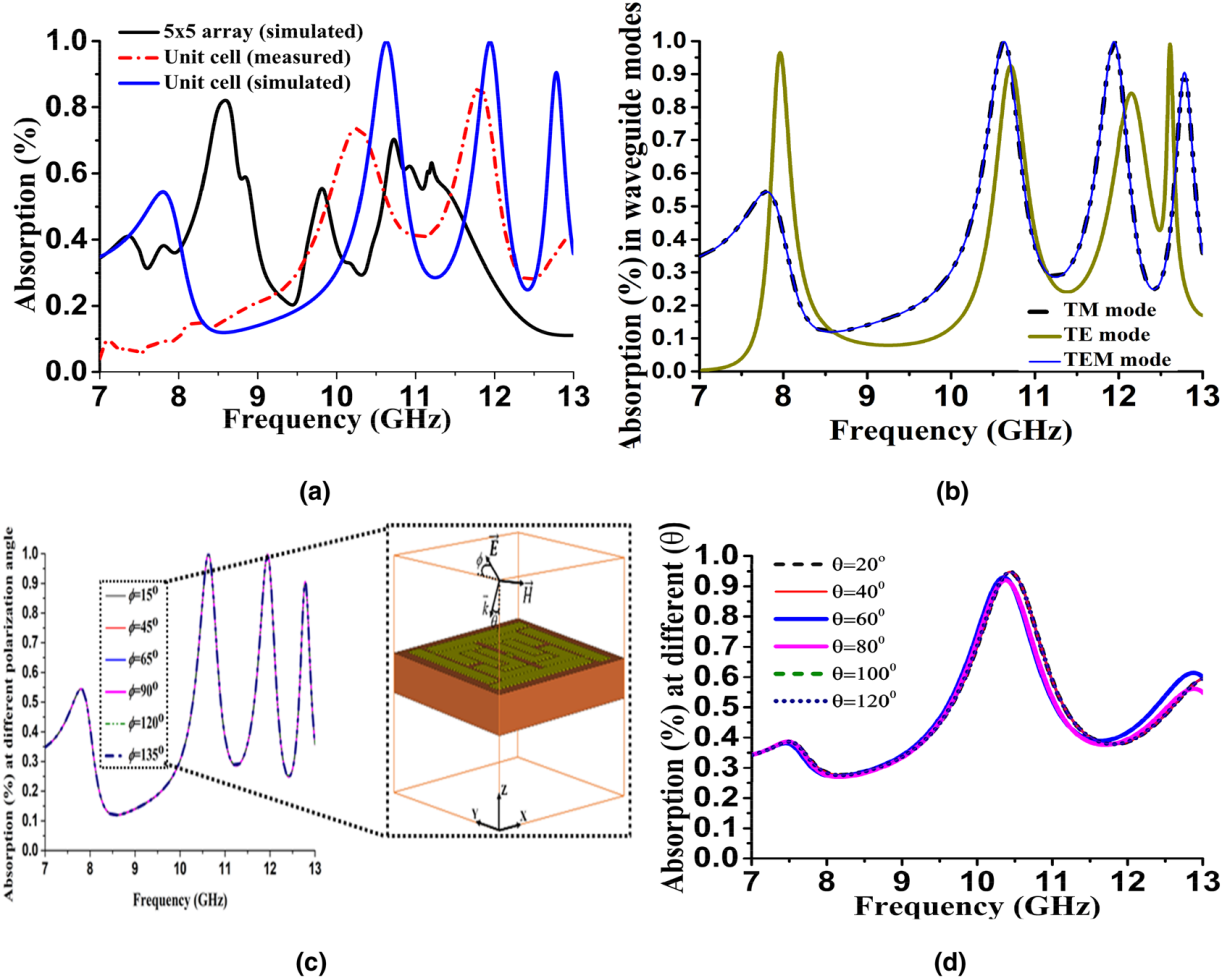
Unit cell absorption performance (**a**) simulated versus measured absorption percentage (**b**) transverse mode (TEM, TM, TE) absorption (**c**) and (**d**) polarization angle impact on absorption (inset: the relation between and in the unit cell).

The performance of the MRMA absorption from a different perspective is illustrated in Fig. 8. The simulated absorption had three consecutive bandwidths (Fig. 8a), whereas the measured results deviated in terms of their absorption percentages and resonance points. During the measurements, the wet cotton and unit cell structure needed to be fixed frequently for proper placement in the waveguide. Nevertheless, the results showed a significant EM wave absorption capacity of the unit cell. Numerical calculations of the reflection loss (RL) for the incident angle were simulated for the TE and TM modes (Fig. 8b). In Fig. 8c, the polarization angle $\phi$ between E-field and Y-axis and wave vector $\vec{k}$ along the Z-axis is defined. The angle of $\phi$ changed from 15° to 135°, while the absorption peak in the triple resonance frequency remained unchanged. The polarization independence of the MRMA is associated with central symmetry in its structure. The incident angle $\theta_{i}$ is defined as the angle between the $\vec{k}$ and Z-axis in the X–Z plane. As $\theta_{i}$ increased, the absorption decreased for the non-reflection coefficient parameter, and the absorption efficiency of the TM polarization was superior to that of the TE polarization. Unfortunately, an incident angle variation on the unit cell (Fig. 8d) showed losses in the absorption percentage (a decline from 99 to 94.75%) and two other resonance frequencies compared to $\phi$. Under normal incidence, the absorber follows the conditions in Eq. (6).

However, the zero reflection condition changed as the angle changed. The impedance between the absorber and free space became mismatched, following the equation of reflection for perpendicular and parallel polarization rather than normal condition^34^. Furthermore, the full width at half maximum (FWHM) of the proposed design were 0.61, 0.51 and 0.28 GHz for *f*_1_, *f*_2,_ and *f*_3_, respectively. Thus, we have a frequency selectivity option with fractional bandwidth. However, the simulated and measured Q-factor, defined as Q = *f*_0_/FWHM, showed a deviation; for example, *f*_2_ simulated Q was 23.43, whereas the measured one was 18.1. For sensor-related application, this Q-factor has much potential, though further analysis must be performed of surface plasmon waves (SPWs) and the collective oscillations of an electron in the metal surface.

**Figure 9:**
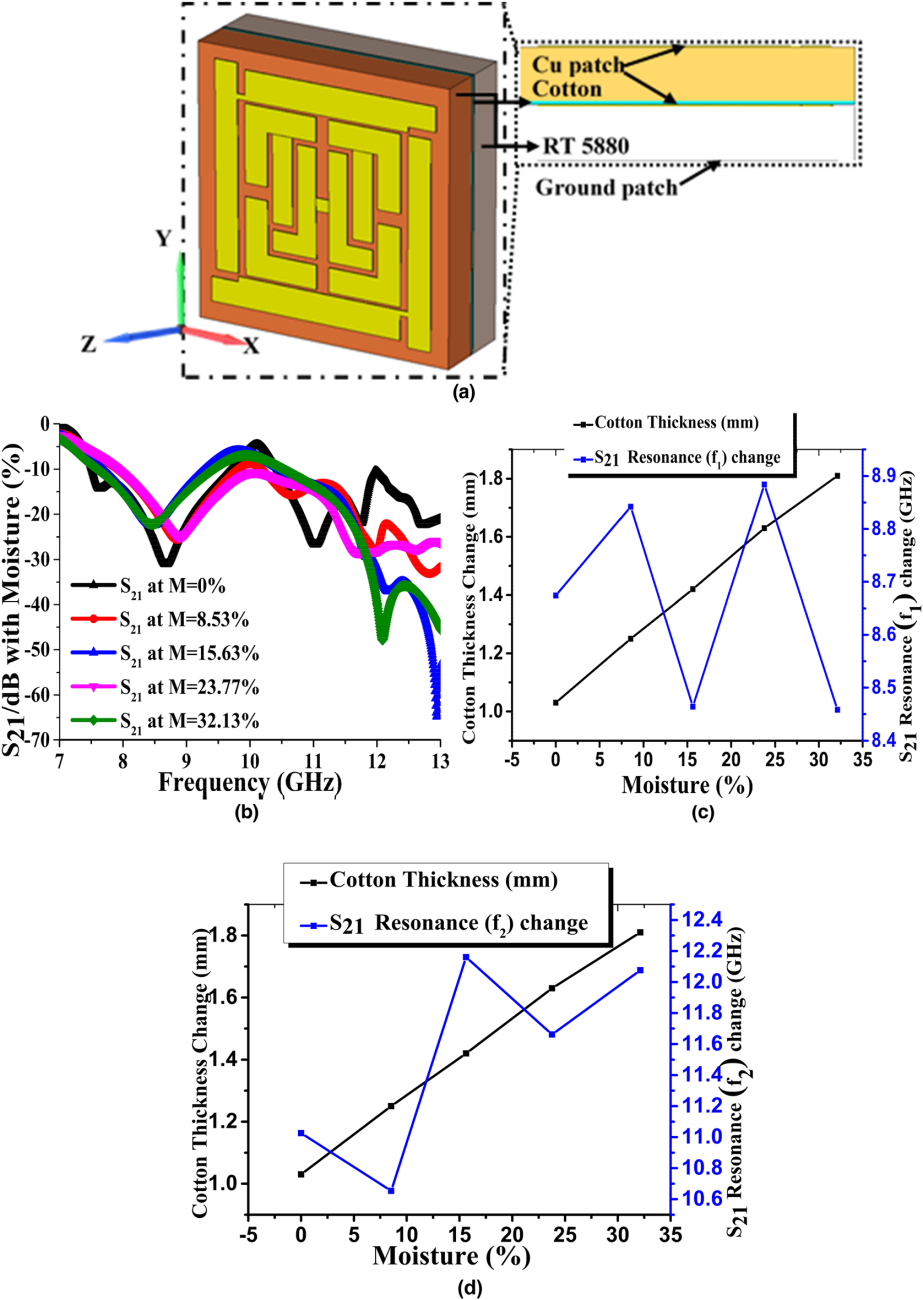
Microwave sensing of moisture using MRMA simulated performance (**a**) microwave sensing of moisture using MRMA (**b**) transmission (S_21_) resonance shifting (**c**) and (**d**) moisture percentage and thickness change on cotton, cotton wetness, and thickness with S_21_ resonance change.

The reason for focusing on cotton in the unit cell was for moisture protection in the form of a bottle of capsules or tablets. Today, most pills are coated; however, the National Institute of Health (NIH)- U.S. Department of Health and Human Services, recommends removing any cotton from inside the bottles, as they could draw moisture into the container. Instead of placing them inside, outside placement and the microwave sensing of possible moisture absorption is a potential method of detecting the moisture level. In addition, other X band sensing applications could present potential applications for the proposed MRMA. The tuning characteristics of the microstructure absorber were estimated by transmission coefficient resonance frequency shifting at different moisture contents (M) (Eq. 11) of the cotton substrate between two-unit cells. *Shifting* refers to the change in the frequency and indicates two specific resonance frequency ranges. The first, *f*_1_, ranges from 8.4 to 8.9 GHz, and the other, *f*_2_, ranges 10.6–12.4 GHz. In the proposed MRMA, the X-axis is considered for electric field and the Y-axis for magnetic field propagation (Fig. 9a). During polarization, at room temperature, the dielectric constant and tangential loss of dry cotton were measured as approximately 4.11 and 0.028, while for wet cotton, they were 15 and 0.031, respectively^35–37^. However, the accuracy of these measurements had a ± 5% deviation. Figure 9b, shows the simulation that was performed starting with dry cotton (M = 0%) and with a wet state (M≈32.13%) as an arbitrary value. It was observed that the *f*_1_ and *f*_2_ resonance points began in the dry state of the cotton at 8.67 and 11.02 GHz, respectively (Fig. 9c,d). In a wet state, *f*_2_ was expected to change at a higher frequency of 12.07 GHz, but the *f*_1_ dropped to 8.45 GHz, which was unexpected. This scenario can be explained through the fact that cotton is anisotropic and has a high moisture content and a highly lossy dielectric at microwave frequency. As it showed unusual behavior, the Debye formula was considered for a reasonable estimation of the moisture (or water) permittivity^38^. The moisture content (M) is defined as

11 $$ M[\% ] = \frac{{m_{2} - m_{1} }}{{m_{1} }} \times 100 $$

where *m*_*1*_ and *m*_*2*_ are the masses of dry cotton and wet cotton, respectively. In this work, 23.13% of the moisture content was used for the wet state. However, the M gradually changed from a dry state (M = 0%) to a wet state 32.13%, with 8.53%, 15.63%, and 23.77%. A gradual increasing change in the resonance frequency (*f*_1_ and *f*_2_) was observed with two or three fluctuating points. However, increasing the number of samples from lower to higher frequencies would provide a clearer picture of the behavior. Further investigation of this prototype will consider this matter. Figure 9b–d indicates that the proposed fabricated absorber had a resonance point shifting as an impact of moisture amount.

Before proceeding with the measurements, it is necessary to discuss metamaterial sensor comparisons, other than those that use an open-ended coaxial probe. The microwave measurement technique has been studied for sensing different phases of materials such as small granular size, liquid, semi-liquid, or solid phases. In general, focusing on our sensing area, the developed method permittivity measurement can be classified as—transmission/reflection line (TRL) method, open-ended coaxial probe method, free space method, and resonant method^39^.

Previous studies have clarified the potential of these methods, as charted in Table 3. For example, liquid sensing has been investigated using an integrated transmission line and has shown significant accuracy in fuel (diesel, gasoline, ethanol, methanol) electrical property characterization^40–42^. The phase shift transmission line demonstrated certain unknown multi-layered structures such as the soil moisture sensor^43^. Although the accuracy of TRL measurements is limited by the air-gap effect and MUT length, it is a multiple of a one-half wavelength in a material. Aqueous adulteration evaluation using microstrip sensor antenna in the free space method has been used to demonstrate the dielectric effects of impurities in substances^44^. A further low-cost solution utilizes paper substrates proposed for similar applications both in the microwave and terahertz spectrum, measured using a spectrometer^45^. Flat and large MUTs, multiple reflection errors, and sample edge diffraction components are a significant limitation of the free space method. Changes in the substrate thickness and dielectric properties have been shown using the resonator method for multi-layered structures^46^. High sensitivity and selectivity featured biosensors in the IR range are another typical example of resonator-based sensors^47^. However, the precision of the results of the resonator method requires a high-resolution VNA and is limited to narrowband frequencies. Here, the proposed unit cell shows more accurate moisture sensing in terms of dielectric property characterization through the coaxial probe method. However, this method is not beyond error-free data extraction, as reflection measurements and the air-gap affect the results.

**Table 3:** Metamaterial sensor application and sensing method comparison other than open-ended coaxial probe.

Ref. paper	Method	Key parameter identification	MUT	Method accuracy	Remarks
^48^	TRL	Complex permittivity (ε) and permeability (*µ*)	Low loss sample	Medium good	Robust algorithm can improve results
^42^	Resonant	S_21_ resonance shifting is the critical point	Methanol–water and ethanol–water	Very good	detection of fluidics
^44^	FSM	S_21_ resonance shifting is the critical point	NaCl and C_2_H_4_O_2_ substances	Medium good	impurities level identity
^45^	Resonant	S_21_ resonance shifting is the critical point	Oil, methanol, glycerol and water	Very good	Dielectric properties
^43^	TRL	Determination of permittivity	Microfluid	Medium good	Phase shift method for calculation

**Figure 10:**
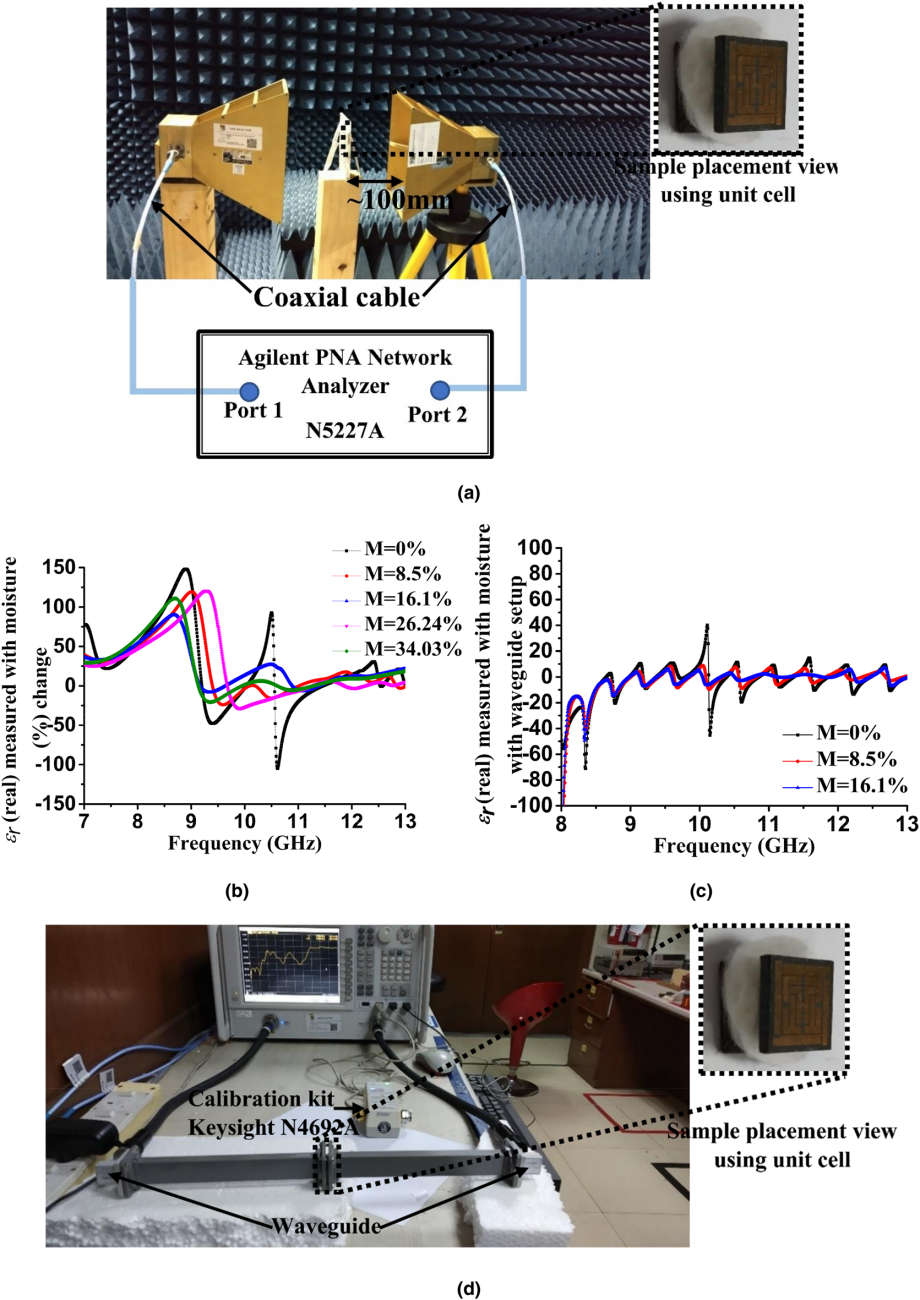
Dielectric constant measurement of cotton using (**a**) FSM, (**b**) real value of *ε*_*r*_ using FSM (**c**) WMM (**d**) real value of *ε* using WMM through transmission reflection measurement.

The justification of moisture content on cotton (anisotropic material) and its effect on the dielectric constant is discussed in the numerical study shown in Fig. 9, where S_21_ has a resonance frequency change with moisture percentage. This has been verified by the two extraction methods shown in Fig. 10. There are few established methods for measuring the dielectric constant of fabric or other such materials^35,39,48^. Based on the available measuring equipment in our research lab, we adopted the FSM and WMM to numerically extract the real value of dielectric constant. To measure moisture in cotton, we used the commercially available Floureon portable digital cotton moisture meter tester MS7100C, which has a measurement capacity of 7–40% with an accuracy of ± 0.5%. Two leads connected to the probe cable were placed at each instant of water drop and recorded the moisture percentage. Five (5) cotton slabs approximately 10 × 10 mm were placed between two conducting leads of meter to try to get exact ratios as follows 0%, 8.5%, 16.1%, 26.24%, and 34.03% for make similarity with the previously mentioned moisture percentages during the simulation.

The FSM shown in Fig. 10a consisted of two horn antennas connected between a PNA and antenna using an extended low-loss coaxial cable. The unit cell MUT was placed between two antennas. Several advantages of FSM have been presented in previous literature, it is contactless, non-destructive, and resistant to harsh environment, such as too high temperature^49,50^. The significant limitations of FSM are the mutual coupling between radiating elements, requiring a large area of MUT for precise calculations and specific antenna movements with perfect system calibration. The Keysight N4692A MW electronic calibration (ECal) module has been used for VNA. ECal accuracy is suitable for network analyzer two-port calibration, reduces the possibility of errors and requires less time and effort compared with traditional mechanical kits. To avoid these problems, use extended the waveguide(W/G) with a W/G coaxial adapter (Fig. 10d), which compressed the microwave energy. This resulted in an improved measuring method that measures the direct wave penetration rather than the spherical nature of the transmitted waves. This allows the distance between the antennas and the sample to be severely reduced without violating the theoretical assumptions (i.e. the no plane-wave condition). As a consequence, the transversal dimensions of the sample can also be reduced, without the use of horn antennas^51^.

Data extracted in WMM were further treated with the developed algorithm to determine the correct roots of the reflection co-efficient (Γ) and transmission coefficient (T), rather than a direct determination of real permittivity (ε_r_)^52^. The imitate algorithm is suitable for Rogers RT5880 based samples, as it has low dielectric loss and low moisture absorption^52^. The ε_r_ (real) shown in Fig. 10b, c extracted values in WMM were much closer to the accurate values. As listed in Table 4, dielectric is constant of respective resonance frequencies. Typically, the *ε*_*r*_ of cotton ranges from 1.3 to 2.33 and is dependent on the processing criteria^35^. Considering this as a reference value, a deviation or fluctuation in both methods of measurement can be observed. However, WMM has much closer range variation compared with FSM, as it considers the *correct root* from the solution, rather than using all roots from S_11_ and S_21_. For example, *f*_*2*_ and *f*_*3*_ have variations of approximately 1.94–12.07 and 1.17–18.33, respectively, whereas FSM gives negative values (− 103.3, − 13.37, − 2.4), which are not acceptable due to the air dielectric effect. Therefore, a closer variation of ε_r_ signifies that a much more accurate measurement of the cotton dielectric constant is possible using the proposed unit cell and proves the sensing capacity in a given moisture value. It is noteworthy that, though the *ε*_*r*_ values for the moisture were 26.24% and 34.03% in FSM, unfortunately, during measurements for WMM with moisture, the unit got direct contact with water and S_11_, S_21_ did not show significant resonance. As a result, the S_21_ became almost incapable of extracting the roots. Hence, these values were not compared in Table 4.

**Table 4:** Relative permittivity (*ε*_*r*_) with moisture (%) change using the FSM and WMM method.

Measurement method	Moisture percentage (%)	*ε*_*r*_ (real) at resonance f_1_ (10.62 GHz)	*ε*_*r*_ (real) at resonance f_2_ (11.64 GHz)	*ε*_*r*_ (real) at resonance f_3_ (12.8 GHz)
FSM	0	− 103.3	5.05	17.99
WMM		− 2.4	1.94	2.12
FSM	8.5	− 13.37	12.07	2.37
WMM		2.82	0.95	1.17
FSM	16.1	22.78	7.56	18.83
WMM		2.44	1.03	− 3.81

The comparative study to similar research, shown in Table 5, is presented to aid in our understanding of the contribution of the MRMA absorber. For example, Ling et al.^53^ reported FR-4 substrate-based absorber with an absorption of over 90% in the X band, whereas this unit cell can achieve a similar absorption amount with a small dimension. Likewise, in Bakir et al. and Abdulkarim et al., a lower absorption rating with a higher dimension of unit cell compared to the proposed MRMA was reported^54–56^. Therefore, in terms of size, bandwidth, and absorption, the proposed unit cell performance was superior to others, and it, therefore, has potential utility in X band applications.

**Table 5:** Comparison between the proposed absorber and related wideband absorber for sensing.

Paper	Absorption (%)	Dimension (mm)	Bandwidth	Substrate	Frequency range (GHz)
^8^	Above 90	12 × 12	Narrowband	FR-4	C and X band
^9^	Above 90	10 × 10	Narrowband	FR-4	X band
^53^	Above 90	33 × 32	Wideband	FR-4	2.7–5.7
^54^	50	22.86 × 10.16	Ultra-narrowband	RT 5,870	X band (8–12)
^55^	Above 80	24 × 30	Narrowband	FR-4	1–6
^56^	Above 80	22.86 × 10.16	wideband	FR-4	X band
Proposed	Above 90	10 × 10	Fractional band	RT 5,880	X band

## Conclusion

In summary, a metamaterial absorber is proposed using a tunable microwave frequency (X band) for moisture sensing. Hygroscopic fiber such as cotton slab is used between two-unit cell MRMA. Its performances, triple fractional bandwidth absorption is above 90% in simulation, whereas approximately it is 80% absorption with two wideband during measurement. The complex structure of the absorber ensures perfect metamaterial property with a minimal value of backward propagation (DNG at 12.8 GHz, SNG at 10.62 GHz, and 11.64 GHz) for modified dielectric characteristics leading to perfect EM absorption. Besides, two RT 5,880 PTFE substrates sandwiched with cotton slab boost the potentiality of application since the combined structure relates a high-frequency microwave field and trending or retain moisture capacity. Besides, simulated moisture sensing depicts two independent resonance frequency changes, *f*_1_ (8.4–8.9 GHz) and *f*_2_ (10.6–12.4 GHz) with moisture level variation on the slab, which might be useful to detect moisture in the capsule container. This nature of the metamaterial absorber may have other potential prospects in a variety of commercial products in the X band, wireless humidity sensors use a low-cost solution.

## Methods

Unit cell developed on two adjacent vertical long patches with a tiny drop hole at the peripheral, inner side square shape patch with four slits and symmetrical L-I patches connected with a small-single strip line. Multipart, well-adjusted shape followed by the transmission line principle shows good resonance in transmission and absorption characteristics. This MRMA has been fabricated on Rogers RT 5,880 (lossy) thickness of 1.575 mm substrate, which is Polytetrafluoroethylene (PTFE) synthetic fluoropolymer. This substrate is ideal for a high-frequency applications like satellite communications and radar systems. The physical geometry of the unit cell is symmetric along the vertical axis and simulated through commercially available CST microwave studio 2017 software.
